# Supplementary figures and images for: Feature blindness: A challenge for understanding and modelling visual object recognition
Source: PLoS Comput Biol. 2022 May 13;18(5):e1009572. doi: 10.1371/journal.pcbi.1009572 (PMC9132323; doi:10.1371/journal.pcbi.1009572)

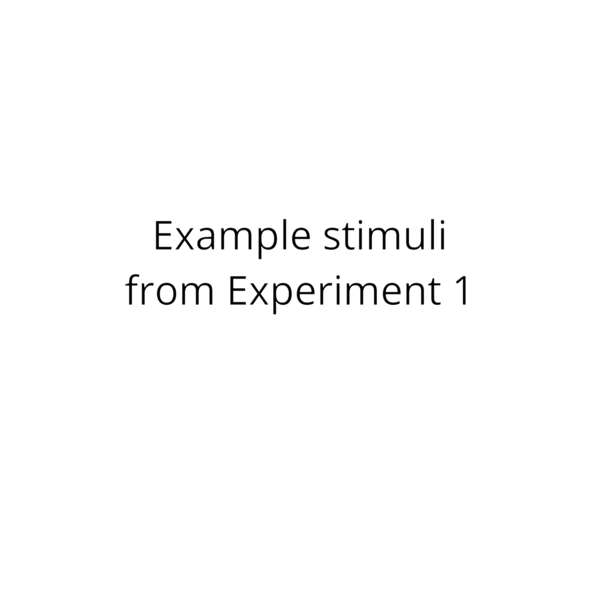

Supplement: S1 Movie — (GIF) [file pcbi.1009572.s001.gif]

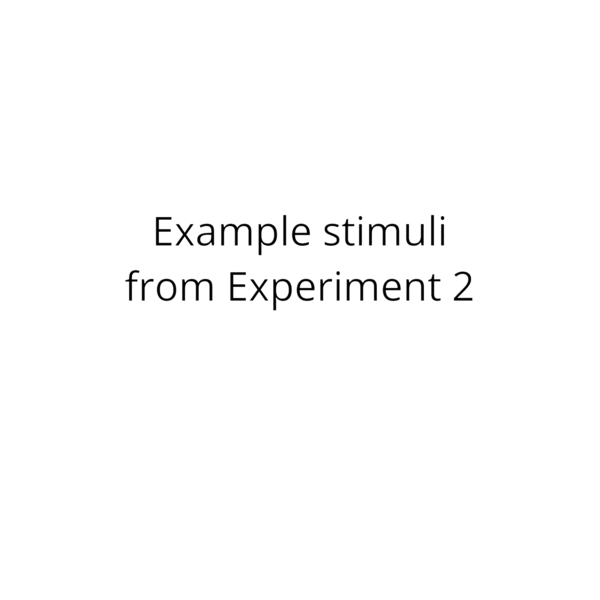

Supplement: S2 Movie — (GIF) [file pcbi.1009572.s002.gif]

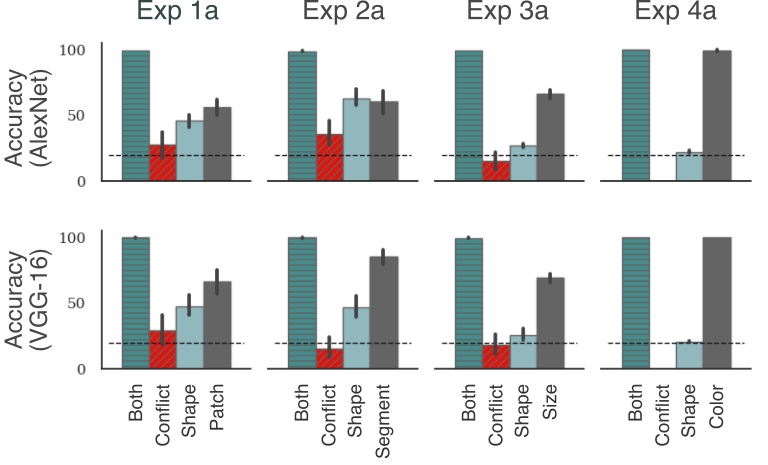

Supplement: S1 Fig — Each panel shows the accuracy under the four test conditions for AlexNet (top row) or VGG-16 (bottom row). Each column corresponds to a different experiment. Both models were pre-trained on ImageNet and fine-tuned by reshaping the final layer to reflect the number of target classes in each experiment and trained on 2000 images from the training set (see Materials and methods for details). A comparison with Fig 3A shows that both architectures showed the same pattern of results as ResNet50: models were able to learn the task (high accuracy in the Same condition), learned both the Shape and Non-shape features (above chance accuracy in Shape and Non-shape conditions) and preferred to rely on the Non-shape feature (low accuracy in the Conflict condition). (TIFF) [file pcbi.1009572.s003.tiff]

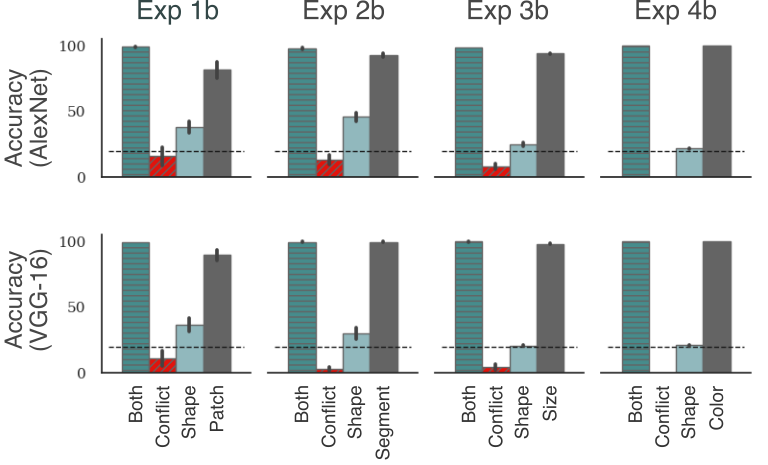

Supplement: S2 Fig — Each panel again shows the accuracy under the four test conditions for AlexNet (top row) or VGG-16 (bottom row). Each column corresponds to a different experiment. A comparison with Fig 3B shows that both architectures showed the same pattern of results as ResNet50: models showed a strong preference to rely on the non-shape feature in this case (a high-low-low-high pattern in the Same-Conflict-Shape-Non-shape conditions) and this preference became larger than the experiments where both features were equally predictive (compare with S1 Fig above). (TIFF) [file pcbi.1009572.s004.tiff]

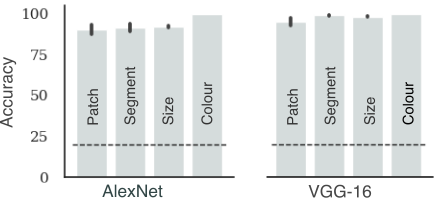

Supplement: S3 Fig — The two panels show accuracy in test blocks for AlexNet and VGG-16, respectively, when these models were trained on images that lack any coherent shape (Experiment 5). Each bar corresponds to the type of non-shape feature used in training. Like ResNet50, but unlike human participants (compare with Fig 5B), both models were able to learn all types of non-shape features. (TIFF) [file pcbi.1009572.s005.tiff]

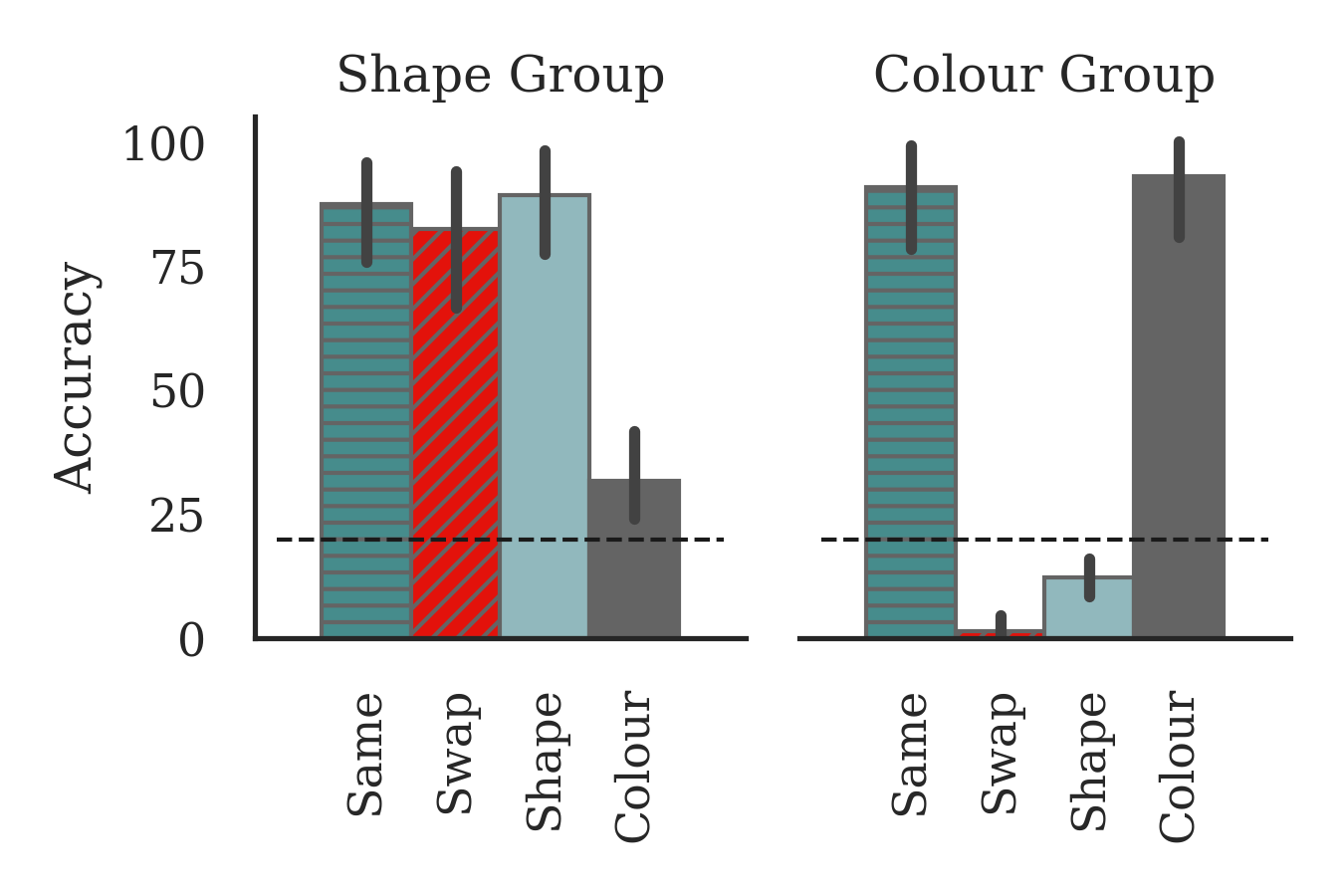

Supplement: S4 Fig — Each panel shows the accuracy under the four test conditions for a subgroup of participants. Participants were split based on whether they performed better in the shape or colour conditions. The first group contained N = 12 participants and the second group contained N = 13 participants. (TIF) [file pcbi.1009572.s006.tif]

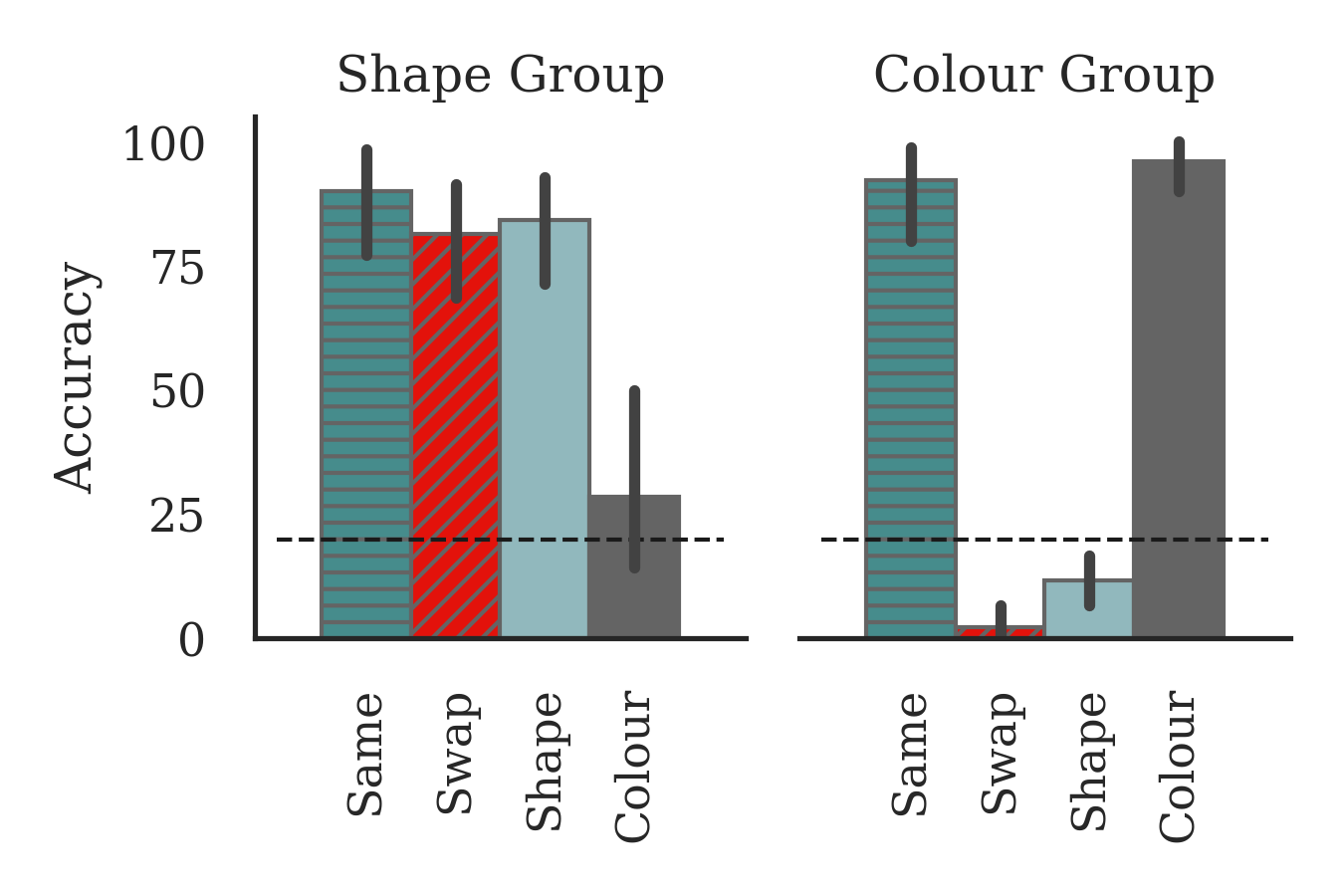

Supplement: S5 Fig — Each panel again shows accuracy under the four test conditions for the subgroups of participants who prefer to rely on shape and colour, respectively. In this case, the first group consisted of N = 7 participants and the second group consisted of N = 18 participants. (TIF) [file pcbi.1009572.s007.tif]

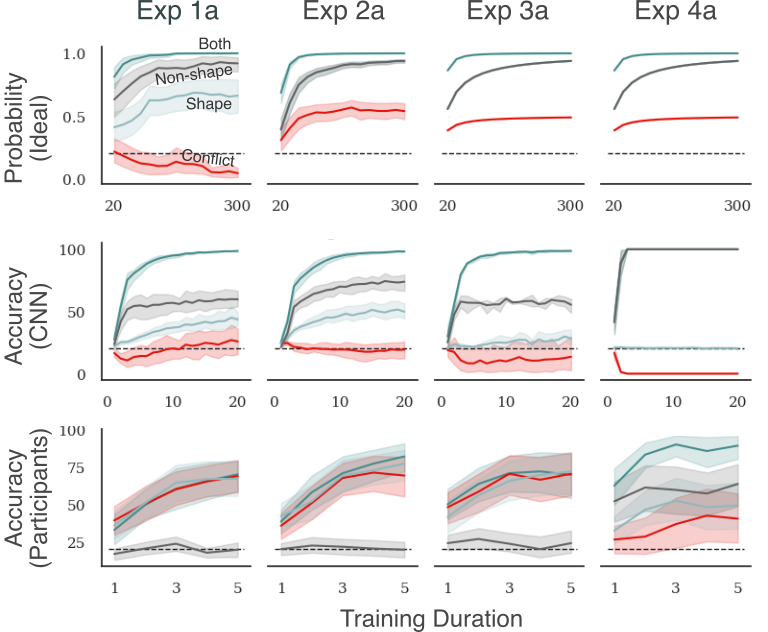

Supplement: S6 Fig — Fig 5A in the main text shows the change in performance under the four test conditions in Experiment 1b, 2b, 3b and 4b, where the non-shape feature and more predictive than shape features in training. Here we have plotted how performance changes in Experiments 1a, 2a, 3a and 4a, where both features are equally likely. Each panel shows how accuracy on the four types of test trials changes with experience. The top, middle and bottom row correspond to optimal decision model, CNN and human participants respectively. Columns correspond to different experiments. The scale on the x-axis represents the number of training trials in the top row, the number of training epochs in the middle row and the index of the test block in the bottom row. A comparison of S6 Fig and Fig 5A from the main text shows a very similar pattern in all experiments and for humans as well as the two types of models. The two models predict that a difference between Both and Conflict conditions emerges early and grows with learning. In contrast, human participants show no difference in the two conditions throughout the experiment in Experiments 1a, 2a and 3a. Further analysis of individual participants showed that, like Experiments 1b, 2b, 3b and 4b, no participant switched from using one feature to another during the experiment. (TIFF) [file pcbi.1009572.s008.tiff]

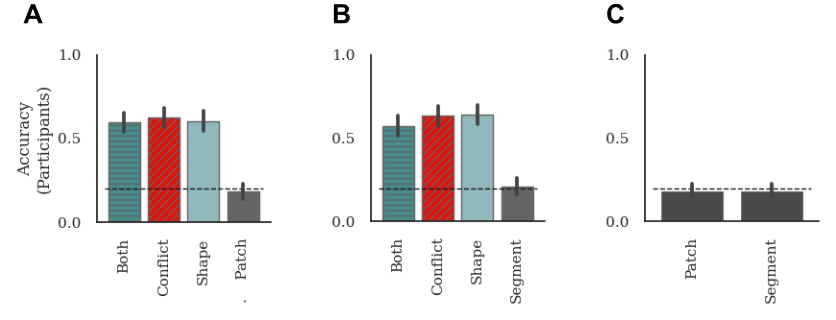

Supplement: S7 Fig — In three experiments, we tested how participant behaviour changed when we presented the stimuli for a shorter duration (100ms) and restricted the field of view, such that the stimulus was always presented within 10° of fixation (see Materials and methods in main text). (a—S7A Fig) Accuracy of N = 25 participants in the four test conditions in an experiment that mirrors Experiment 1b—i.e., all training images contain a diagnostic patch and 80% images contain a diagnostic shape, (b—S7B Fig) Accuracy of N = 25 participants in the four test conditions in an experiment mirroring Experiment 2b—i.e., all training images contain a diagnostic segment and 80% images contain a diagnostic shape, (c—S7C Fig) Accuracy of two groups of N = 10 participants in test block where the training images contained only non-shape cues. Performance of participants in all experiments was consistent with their performance observed in other experiments. Though the overall accuracy of participants in this control experiments was slightly lower (mean accuracy in the Both condition was M = 59.60% in (a) and M = 57.20% in (b)), which is understandable given the faster presentation time, there was statistically no difference in their performance in the Both, Conflict and Shape conditions and their performance in the Non-shape condition was at chance. In Experiment (c), where there was no shape features in the training set, performance of both the Patch and Segment groups was statistically at chance. That is, participants consistently learned based on shape cues; when a diagnostic shape was not present during training, no participant managed to learn the task. (Compare results with Figs 3B and 5B). (TIFF) [file pcbi.1009572.s009.tiff]

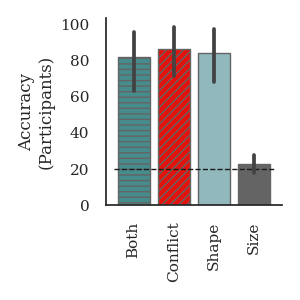

Supplement: S8 Fig — Accuracy in the four conditions when participants are shown the stimuli for 3s instead of 1s. In this experiment, every trial has two diagnostic features—global shape and average size. Despite the increase in the duration of the stimulus, participants performed well in the Both, Conflict and Shape conditions, but performed at chance in the non-shape (Size) condition, indicating that they still preferred to learn based on shape. Notice, we used Experiment 3 (non-shape cue = average size) to test this because this is experiment in which the participants were most likely to pick on the non-shape (Size) cue based on results in Experiment 5, where mean performance in the Size condition was above chance, while mean performance in Segment or Patch conditions was at chance, even when there was no competing shape feature. (TIFF) [file pcbi.1009572.s010.tiff]

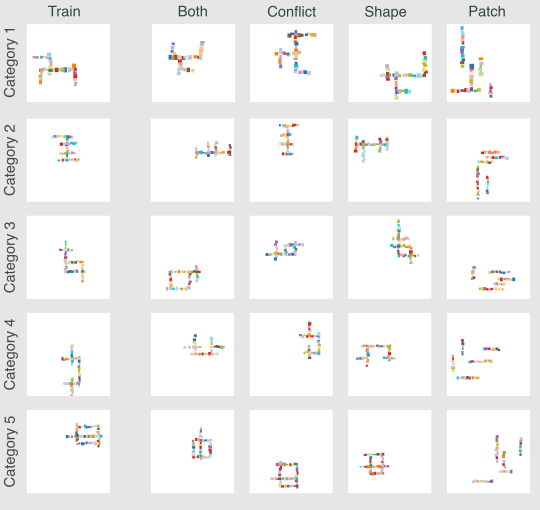

Supplement: S9 Fig — In each row we show (from left to right) an example image from the training set, Both condition, Conflict condition, Shape condition and Non-shape (Patch) condition for a category. Each image in the training set contains a diagnostic patch of a certain colour that is present at a category-specific location. Additionally, all training images in Experiment 1a and 80% of images in Experiment 1b have a diagnostic shape. Images in the Both condition contain both these features. Images in the Conflict condition contain the shape from one category but diagnostic patch from another category. Images in the Shape condition contain the shape feature but none of the diagnostic patches. Images in the Patch condition contain the diagnostic patch but none of the shapes from the training set. (TIFF) [file pcbi.1009572.s011.tiff]

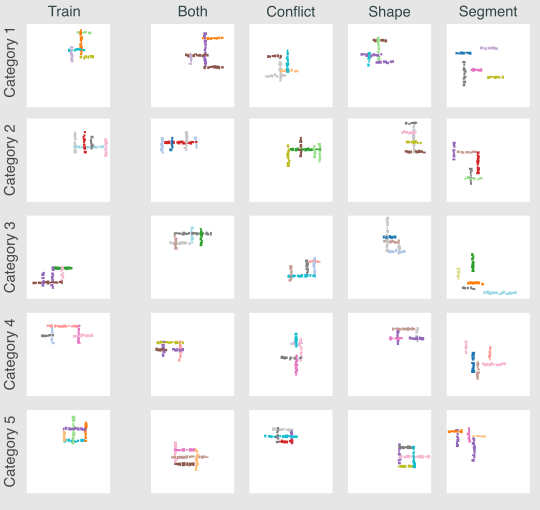

Supplement: S10 Fig — In each row we show (from left to right) an example image from the training set, Both condition, Conflict condition, Shape condition and Non-shape (Segment) condition for a category. Each image in the training set contains a diagnostic segment of a category-specific colour. Only images of this category have a segment of this colour. Additionally, all training images in Experiment 2a and 80% of images in Experiment 2b have a diagnostic shape. Images in the Both condition contain both these features. Images in the Conflict condition contain the shape from one category but diagnostic segment from another category. Images in the Shape condition contain the shape feature but none of the diagnostic segments. Images in the Segment condition contain the diagnostic segment but none of the shapes from the training set. (TIFF) [file pcbi.1009572.s012.tiff]

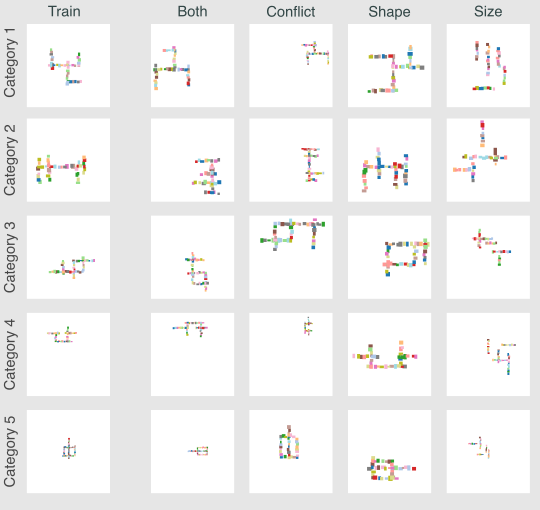

Supplement: S11 Fig — In each row we show (from left to right) an example image from the training set, Both condition, Conflict condition, Shape condition and Non-shape (Size) condition for a category. The average size of all images in the training set is diagnostic of the category. That is, different categories have images that have different average size of patches. Additionally, all training images in Experiment 3a and 80% of images in Experiment 3b have a diagnostic shape. Images in the Both condition contain both these features. Images in the Conflict condition contain the shape from one category but diagnostic size from another category. Images in the Shape condition contain the shape feature and the average size of patches is larger than the diagnostic size of any category in the training set. Finally, the Size condition contains images where the average size of patches is diagnostic but shape is not. (TIFF) [file pcbi.1009572.s013.tiff]

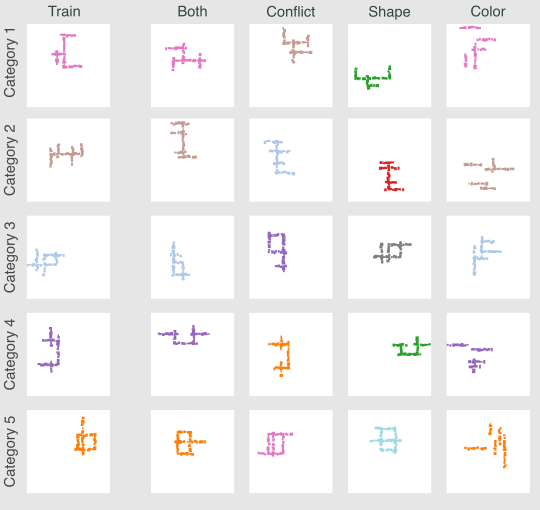

Supplement: S12 Fig — In each row we show (from left to right) an example image from the training set, Both condition, Conflict condition, Shape condition and Non-shape (Size) condition for a category. All patches in an image have the same colour. This colour is diagnostic of an image’s category in the training set. Additionally, all training images in Experiment 4a and 80% of images in Experiment 4b have a diagnostic shape. Images in the Both condition contain both these features. Images in the Conflict condition contain the shape from one category but diagnostic colour from another category. Images in the Shape condition contain the shape feature and a colour that is not diagnostic of any category in the training set. Finally, the Colour condition contains images with no coherent shape but where the colour of segments is diagnostic of the category. (TIFF) [file pcbi.1009572.s014.tiff]

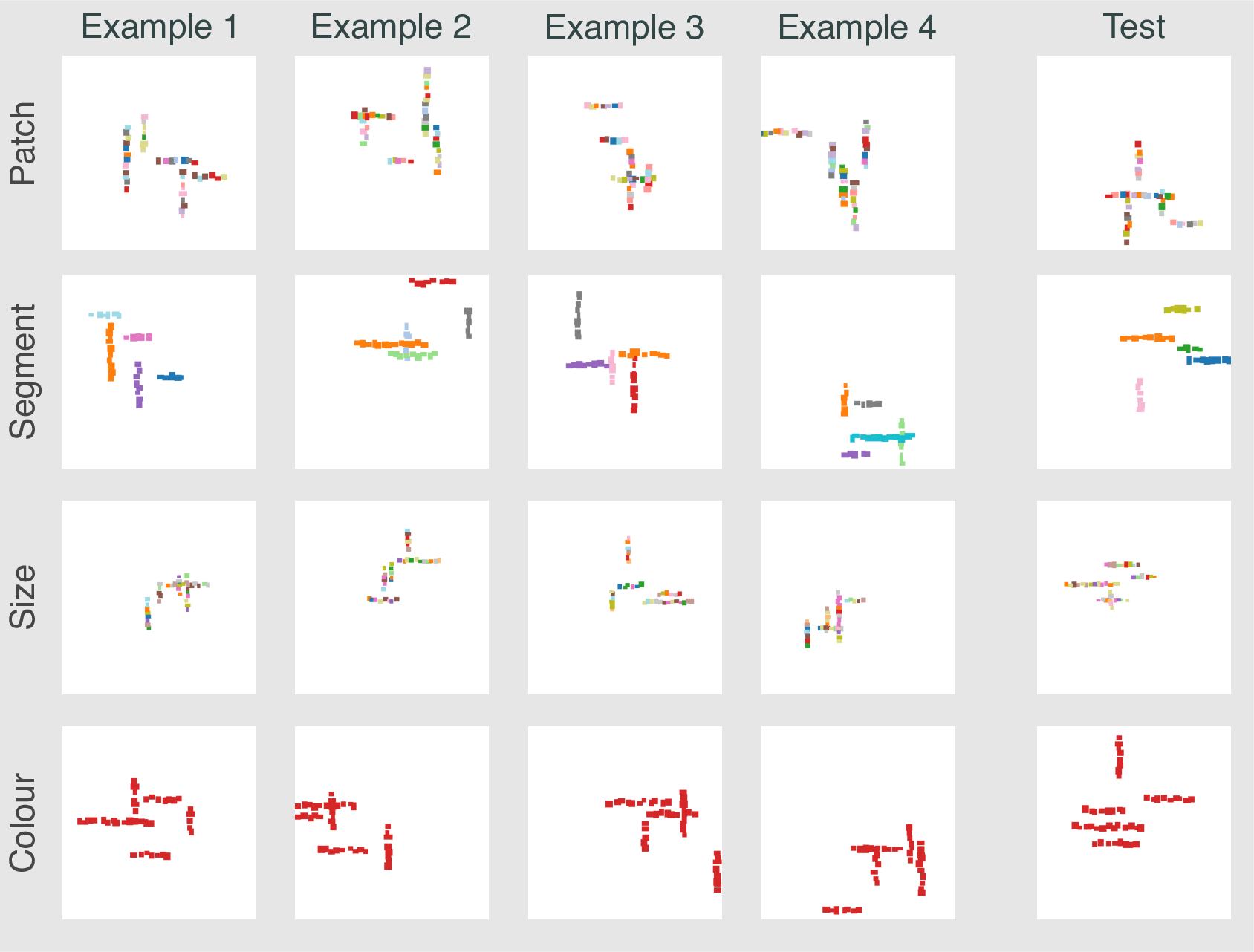

Supplement: S13 Fig — Each row shows four examples from the training set that have the same category label as well as one example from the test set with the same label. The four rows correspond to the four conditions. In row 1, the predictive feature is patch location. In row 2, the predictive feature is colour of one of the segments. In row 3, the predictive feature is average size of patches. And in row 4, the predictive feature is colour of all patches. (TIF) [file pcbi.1009572.s015.tif]
