# Supplementary material for: Feature blindness: A challenge for understanding and modelling visual object recognition
Source: PLoS Comput Biol. 2022 May 13;18(5):e1009572. doi: 10.1371/journal.pcbi.1009572 (PMC9132323; doi:10.1371/journal.pcbi.1009572)
Supplement: Attachment — Submitted filename: Response_Letter_final_PCOMPBIOL-D-21-01905.pdf [file pcbi.1009572.s016.pdf]

Dear Drs. Blake A Richards and Samuel Gershman:

We would like to start by thanking the editors for their extremely useful advice on how to improve the manuscript. It is rare to get such a detailed response from editors and we really appreciate the time spent by the editors and the thoughtful feedback they provided. We would also like to thank all the reviewers for their feedback. We understand that while reviewers in general liked the experiments, they felt the conclusions drawn were stronger than the ones warranted by our observations. One reviewer was also concerned about the size and duration of stimuli used in the experiments. As a result of this feedback, we have made two key changes to the manuscript:

1. We have revised the Introduction as well as the Discussion of the paper to more accurately frame the goals of our study and the conclusions we can draw from it. We had previously concluded that our results show that human behaviour in our experiments is not in line with CNNs and statistical inference models. Reviewers pointed out that this conclusion is not warranted based on our data. Upon reflection, we agree that this conclusion is too strong. While our data provides clear difference between participant behaviour and the models we tested, it is still possible that there are other statistical models (and CNNs) that may be able to explain participant behaviour. Moreover, reviewers have pointed out that several studies (Geirhos et al, 2018, Hermann, et al, 2020) already show that CNNs struggle to show a shape-bias and have asked what our study adds to these known findings. In response to this feedback, we have clarified that our finding is *not* that humans show a shape-bias while CNNs don't – both those observations have indeed been made previously – but that humans fail to adapt to a new learning environment consisting of hundreds of trials where a novel feature is perfectly predictive and clearly visible. It is not clear how to build in this "inflexibility" of learning into CNNs. Indeed, solutions suggested for introducing shape-bias to CNNs in previous studies, such as pre-training networks on different datasets (Geirhos et al, 2018 and Hermann et al, 2020), are inadequate for this.
2. We have carried out three new experiments where, as suggested by the reviewer, we reduced the presentation time (to 100ms), decreased the field of view (to 10 degrees) and increased the number of trials available for the participant to learn (to 450 trials). Despite these changes, we observed the same pattern of behaviour by participants as previously reported in our studies – when stimuli simultaneously contained shape and non-shape features participants still chose the shape feature to categorise stimuli (even though the non-shape feature was more predictive in these experiments). When we completely eliminated the shape feature, so that stimuli only contained predictive patch location / segment colour, participants struggled to learn the task entirely. We have added these new results to Appendix D.

In addition to these changes, we have also made a number of other changes to manuscript in response to the feedback provided by the reviewers (all major changes are highlighted in blue in the marked copy). Below, we respond in detail to all comments by the editors and reviewers.

Best wishes,

Gaurav Malhotra, Marin Dujmovic, and Jeffrey Bowers.

****

RESPONSES (interleaved):

****

On 2021-12-08 17:33:21+00:00
em.pcompbiol.0.77dd17.d9243747@editorialmanager.com wrote:
Dear Dr. Malhotra,

Thank you very much for submitting your manuscript "Feature blindness: a challenge for understanding and modelling visual object recognition" (PCOMPBIOL-D-21-01905) for consideration at PLOS Computational Biology. As with all papers peer reviewed by the journal, your manuscript was reviewed by members of the editorial board and by several independent peer reviewers. Based on the reports, we regret to inform you that we will not be pursuing this manuscript for publication at PLOS Computational Biology.

Though the reviewers recognised that the experimental design was creative and appreciated the in-depth comparison between human and CNN behaviour, two of the three reviewers felt that this study does not help us discriminate between existing models, provide new paths forward for better models of human vision, nor provide sufficient new insights into human visual processing. In particular, it is already known that CNNs show differences to humans, e.g., they rely less on shape than humans, so that aspect of the results is a replication of this known phenomenon. A replication study itself would be fine, but that was not the goal here. This paper sought to make a stronger claim, namely that since humans tend to rely on global features like shape even when there is a stronger predictor in the data they were given in these experiments, then human vision cannot be based on statistical learning/inference. But, as the reviewers noted, this study does not actually demonstrate this.

****RESPONSE**: We acknowledge that some of the conclusions we made in our manuscript were stronger than warranted by our observations, and we have tried to fix this error in the revised manuscript (see the revised Introduction and Discussion). But we also wanted to note here how our findings go beyond previous studies that have observed that CNNs rely less on shape than humans. Some researchers have shown that CNNs have a texture bias when trained on well-known datasets, such as ImageNet, but a shape-bias when trained on datasets that contain a shape-bias. For example, Geirhos et al (2018) have developed a Stylized ImageNet and they show that training on this dataset leads to a larger shape-bias. Similarly, Hermann et al (2020) suggest various data augmentation methods and training parameters that lead to a shape-bias in CNNs. According to these studies, the difference between human and CNN object recognition may lie in their different task environment, rather than in a different mechanism of selecting features. Indeed, Hermann et al write: “Our results indicate that apparent differences in the way humans and ImageNet-trained CNNs process images may arise not primarily from differences in their internal workings, but from differences in the data that they see”.

In the context of these studies, our study shows that human adults prefer to categorize visual stimuli based on shape (and other global features) *even when shape is not the most predictive feature in the learning environment*. Thus, unlike Geirhos et al, Hermann et al, and Feinman & Lake findings with CNNs, it does not seem necessary for humans that shape is the most predictive feature of object categories in the current environment for them to show a shape-bias. Of course, we acknowledge that our study is limited by the fact that this learning environment is a psychological experiment consisting of a few hundred trials and our participants are human adults. It is possible that participant behaviour is driven by their experience as infants where their learning environment may indeed have contained a

shape-bias. We have revised our Discussion to make this clear (see, for example, lines 459 – 471). Nevertheless, it is an important observation that human adults do not fully adapt to the new learning environment and start categorizing stimuli based on whatever feature is most predictive in the new learning environment. In contrast, we show that CNNs do adapt to the new learning environment and there doesn't seem to be an obvious way to prevent them from doing this. For example, we show that past training on a dataset containing a shape-bias does not help and neither does decreasing the learning rate or even entirely freezing the weights of all convolution layers after introducing a shape-bias through pretraining. We have also clarified that the statistical inference model serves as a benchmark for model that learns solely based on information present in the experiment and no priors (see, for example, lines 77 – 85, in the revised Introduction). It may be possible to build in priors in the statistical inference model to prevent it from learning based on the most predictive feature, but it is not clear whether these priors will be able to account for the behaviour of participants, especially in Experiments 5 and 6, where no concurrent shape feature is present, and participants still fail to learn based on a predictive feature. Therefore, the tasks and datasets created in our study provide a set of important observations that can help to design and tease apart future models of human object recognition.

First, there are numerous differences between CNNs and humans (and their training) that could potentially explain the differences in performance on the tasks investigated here and which were by no means controlled for, and thus, we can't really say based on the data here what is causing the difference.

****RESPONSE:** We have now carried out three new experiments that control for the key differences in human experiments and CNN simulations highlighted by Reviewer#3. We reduced the presentation time and controlled the field of view in line with the parameters mentioned by Reviewer#3. The same pattern of results was obtained: participants preferred to use shape to non-shape features, even when the non-shape features were more predictive (Experiments S1, S2), and when shape features were absent, participants struggled to learn the category mapping (Experiment S3). Of course, this does not mean that we have controlled for *all* differences between human experiments and CNN simulations, but our study shows that our findings are robust across multiple CNN architectures, a range of hyper-parameters, different types of pre-training and different types of predictive features (patch location, segment colour, patch size). While we believe that there is unlikely to be a set of experiment conditions that will make humans behave like CNNs, we acknowledge that we do not control for all possible differences in the experimental setup (see lines 451 – 458 in the revised Discussion).

Second, we cannot conclude from this experiment that humans do not rely on statistics for learning (nor that evolution didn't). For example, as Reviewer 3 noted, there is no way to simulate a real "zero experience" for humans given that the human visual system is pre-trained via evolution and development and humans have a lifetime of experience of interacting with objects in a 3D world that is reflected in their neural representations. As such, it could be that the amount or sort of data provided to the participants in this study was insufficient to overcome a strong bias towards global properties like shape that emerged from a lifetime (and evolutionary history) of such properties being statistically reliable predictors.

****RESPONSE**: Arguing that humans do not rely on statistics for learning in new environments is neither a conclusion that we intended to make, nor a conclusion that is warranted based on our observations. It is for this reason that we had said in the Introduction: "*While performing statistical inferences is certainly important*, models of vision must also consider the cognitive costs and biases in order to be realistic theories of human object recognition." It is indeed possible that there may be another statistical model that has the right sort of priors (perhaps due to evolution) that brings it more in line with participant behaviour. And part of our goal here is to motivate the search for such a model. We think this will be a challenging task, especially in view of our observations in Experiments 5 and 6, where there was no competing shape cue (Experiment 5), and participants were even told what feature to look for (Experiment 6) and they were still not able to learn the task based on a patch or segment colour.

But clearly, we did not clarify this position sufficiently. Therefore, instead of concluding that participants are not purely relying on statistical inference, we conclude that “one cannot explain human behaviour using a simple statistical model that infers the category of a test stimulus based solely on the evidence observed in the training trials and no prior biases. We also found that human behaviour was inconsistent with the behaviour of CNNs as the predictive value of features play a key role in how a CNN learns to classify novel objects. Unlike human participants, previous biases of the network (either learnt through training or built-in through architectural constraints) were not sufficient to overcome this reliance on predictive features. If humans indeed learn in novel environments through a process of statistical learning, these results motivate an exploration of why humans do not quickly adapt to the novel environment in the same way the statistical models presented in this study do. Note that this may be a challenging problem to solve for CNNs and statistical inference models as in Experiment 5 and 6 participants show a bias even when there is no concurrent shape feature.”

Altogether, given these reviews and the considerations they raise, we unfortunately do not believe that this paper is suitable for publication at PLOS Computational Biology.

The reviews are attached below this email, and we hope you will find them helpful if you decide to revise the manuscript for submission elsewhere. We are sorry that we cannot be more positive on this occasion. We very much appreciate your wish to present your work in one of PLOS's Open Access publications.

Thank you for your support, and we hope that you will consider PLOS Computational Biology for other submissions in the future.

Sincerely,

Blake A Richards
Associate Editor
PLOS Computational Biology

Samuel Gershman
Deputy Editor

****
Reviewer's Responses to Questions

Reviewer #1:

In this study, the authors explore the relative biases towards colour and shape in humans and deep convolutional neural networks using a supervised category learning task. They use some inventive new stimuli in which elementary colour patches are composed into segments, which are then assembled into an object.

The major findings are 1) that when global shape is predictive of the category, humans tend to ignore a colour cue that is either a single element or a segment of the object, but use the colour cue if it covers the entire object;

2) that CNNs, by contrast, prefer to use the colour patch, especially when it is made more diagnostic, by including 20% of shape "catch" trials [experimental series B]; 3) that pretraining on the style-transfer version of ImageNet developed by Geirhos et al increases the bias towards shape in the CNN, but not to human levels; that humans cannot use the colour patch or segment even if colour is non-diagnostic, and even if they are told about it.

I liked several things about this report. I thought the stimuli were creative and had the potential to be useful tools for future experimentation.

****RESPONSE:** Thank you for your positive comments.

I appreciate the in-depth attempt to compare humans and CNNs on a novel task.

****RESPONSE:** Again, we appreciate this.

The study is elegantly executed and reported. I like the factorial design in which cues were either presented singly or together (coherently or in conflict). The writing is for the most part clear (except for some omitted/buried details about the task).

****RESPONSE:** Note, we have added Figures 2 and 7 to clarify details of the task.

The question I asked myself whilst reading was what exactly we learned from the results that was new. It is well known in the category learning literature that 1) humans exhibit a shape bias and 2) they struggle to solve high-dimensional classification tasks with novel stimuli and no curriculum. For example, for the latter see the Demons experiments in Pashler & Mozer 2013; there are numerous other examples. It's also been shown a number of times that when multiple cues predict a category label, humans can easily overlook one, even if it is quite salient. Schuck (2015, Neuron) is one example. The human findings here seem to replicate these results, albeit with a new task and stimuli. They do so elegantly, but I found it hard to be surprised by any of the human results that the authors report.

****RESPONSE:** Thank you for this feedback. We agree that there are several parallels between our studies and previous psychological research, such as the studies mentioned by the reviewer. However, we believe that our experiments contain a number of insights that are both novel and important for understanding how humans learn to recognise objects. We should have done a better job in citing this literature and clarifying the contributions of our research, and we have revised the Abstract, Introduction and the Discussion of our paper to correct this error. Here we respond to the specific points mentioned by the reviewer.

Some of our observations may indeed be summarised as a shape-bias. However, shape-bias studies have been primarily concerned with (a) whether children and adults show a shape-bias (e.g. Smith, Landau & Jones, 1988), or (b) how shape bias develops over time (e.g. Smith et al, 2002; Samuelson, 2008; Colunga & Smith, 2008). In contrast, we are concerned here about how shape-bias, once it has been learnt, influences the learning of novel categories. It is for this reason that our paradigm is quite different from the standard paradigm used to study shape-bias. In the standard version of the task (e.g. Smith, Landau & Jones, 1988), children are presented with a novel object with shape and non-shape attribute. Then, they are presented with a test object that matches the original object in either the shape or non-shape feature and asked to label the object. The shape-bias finding is that children prefer to give the same label to the test object that matches the original object in shape over test objects that match the original object in other non-shape features such as texture, colour or size. In contrast to this paradigm, we (a) conduct our study on adults who have already acquired a shape bias, (b) manipulate their environment so that the environment favours learning based on a non-shape feature (Experiments 1-4), or (c) presents a category learning task where shape cannot be used to learn the task at all (Experiment 5&6). We observe that adult participants show an inflexibility in their learning and their shape-bias perseveres even though the environment favours learning based on a different feature. This behaviour contrasts with the way in which CNNs learn novel objects. We don't know of any shape-bias study that investigates these issues (particularly the contrast with neural networks). We have re-written our Introduction to clarify our motivations. We have also re-written our Discussion to clarify the link between our study and typical shape-bias studies (see lines 459 – 472 and 482 – 493).

Similarly, it is true that there are parallels between our studies and some studies in the category learning literature mentioned by the reviewer. However, a central difference is that here we are interested in how shape-bias influences feature selection on a new task. Like us, Schuck et al (2015, Neuron), participants in our study also overlook some cues. However, in the context of understanding object recognition, it is informative to observe that these cues are always non-shape cues and not shape cues (Experiments 1-3). Additionally, we show that participants also overlook these cues even when there aren't multiple cues present - i.e., there is only one diagnostic cue in the task (Experiments 5 & 6). Moreover, unlike Schuck et al (2015), where participants had already learned to perform the task based on one feature, participants in all our experiments had no prior learning on the task and were not told that the task could be learned based on overall shape. Despite this, they gravitated towards some features, such as overall shape (Experiments 1-3) or colour (Experiment 4) and ignored the other features.

And while we do not use a curriculum to facilitate the learning of some features, in contrast to the Demons task of Pashler & Mozer (2013), the features in our task are highly salient. For example, in their Experiment 4 (Difficult condition) the stimuli in the two categories differed by less than 10 pixels on average. In contrast, in our experiments like Experiment 2, 5 & S3, where the non-shape feature was the colour of a segment, this feature was akin to Pashler & Mozer's initial value of the Fading condition, where there was a distinct difference in the horn height of the Demons. In that experiment, Pashler &

Mozer observed that participants were able to learn the task despite the high dimensionality of the task. Thus, the novelty of our study lies in the observation of how a shape-bias constrains learning in new task settings and how highly salient non-shape features are ignored even when they are highly predictive or even the only feature that can be used to learn the task (Experiments 5 & 6). Critically, this behaviour contrasts with CNNs, which have been claimed to learn a shape bias through training (see new Introduction).

Another difference between our study and these previous studies is highlighted by the results of Experiment 6. Here we told the participants what feature to look for and we still saw that participants struggled to learn based on patch location and segment colour. We interpret these results in terms of the limitation in cognitive resources in using these features for categorisation rather than in the discovery of features.

The comparison to CNNs is instructive, but also follows a tradition which has shown that CNNs struggle to recognise global ensembles but are good at using local diagnostic elements (or textures) to solve classification problems. This is perhaps not surprising when you think about their architecture. The convolutional filters learned at each local image location are shared automatically across visual space. This means that they are given a very strong inductive bias to learn position-invariant local cues.

****RESPONSE**: This is indeed a good explanation for the texture bias observed in CNNs in previous studies such as Geirhos et al (2018) and Hermann et al (2020). However, we do not think this explains some of our results. In Experiment 1, for example, the feature selected by the CNN is the location of a single patch, which (unlike a repeating texture) is *not* position-invariant. While both the colour and location of the diagnostic patch were diagnostic in the reported experiment, further simulations showed that the CNN has no problem picking on a diagnostic location even when the colour was made non-diagnostic (that is, all categories had the predictive patch of the same colour). If CNNs learned features that were position-invariant, then they would not be able to pick a position-specific feature, like the one used in this simulation. Thus, in one sense, the CNNs have been given a bias *against* picking this position-specific feature (through weight-sharing) but still prefer it to learning shape. We haven't included this detail in the manuscript as it is somewhat off-topic, but if the reviewer feels it is important, we will be happy to revise the manuscript to include this information.

By contrast, shape and other global features of an array have to be learned in the final layers where disparate interactions across the entire image can be computed. CNNs are built with the wrong inductive bias to recognise shapes. Papers by Bob Geirhos and Sam Ritter, which the authors cite, have made this point before.

****RESPONSE:** Our intuitions are indeed very much in line with the reviewers – we also think that CNNs are built with the wrong inductive bias to recognise shape. However, neither of these papers make this point. First of all, it is important to note that the papers by Geirhos et al (2018) and Reitter et al (2017) come to *opposite* conclusions about shape-bias. While Geirhos et al show that standard CNNs, trained on ImageNet, show a texture bias, Ritter et al argue that they show a shape-bias. Here is what Ritter et al, (2017) writes in the first line of their discussion: "we found that both IB and MNs trained on

ImageNet display a strong shape bias." And here is what Geirhos et al, (2019) write: "We show that ImageNet-trained CNNs are strongly biased towards recognising textures rather than shapes". Our results agree more with Geirhos et al. than Ritter et al. as we also find that networks prefer to rely on non-shape features when both features are present in the dataset.

Moreover, note the phrase 'Imagenet-trained CNNs' used by both sets of authors. This is a crucial aspect of these studies. Both of these studies are trying to establish whether training on Imagenet confers the right type of bias to the networks. This is different from the reviewer's intuition that networks have the wrong type of inductive bias for learning shape. In fact, Geirhos et al suggest that one way of fixing the shape-bias problem, is to develop an augmented training set (like the Style-transfer Imagenet) where texture is no longer diagnostic. Once the network has been trained on such a training set, Geirhos et al find that they start showing a shape-bias, leading them to conclude that "texture bias in current CNNs is not by design but induced by ImageNet training data". In an extension of this study, Hermann et al (2020) also make a similar point, showing that the network *does* develop shape-bias when the training environment is right, concluding that "Our results indicate that apparent differences in the way humans and ImageNet-trained CNNs process images may arise not primarily from differences in their internal workings, but from differences in the data that they see''.

In the context of these studies, our experiments make a different point. Like the reviewer, our intuition is that CNNs have the wrong inductive bias to learn shape. This is why, even when the CNN is pretrained on the training set developed by Geirhos et al, it still prefers to categorise stimuli based on non-shape features (Figure 4 in our study). Thus, our study shows that there is more to human shape-bias than the training environment itself. When humans learn in an environment where non-shape features are more predictive, they still continue relying on shape. This is an important observation and is currently missing from the discussion around shape-bias. Now, it is possible that experience as infants in environment is what is responsible for the origin of shape-bias in humans and some studies (e.g., Smith et al, 2002; Samuelson, 2002) suggest this. However, the new finding here is that, when learning new objects in environments that do not support learning based on shape, humans continue to exhibit shape bias, at least for a few hundred trials. This behaviour is difficult for CNNs to replicate.

I also had trouble understanding many aspects of the report, at least at first. Perhaps these could be clarified for other readers.
- I initially assumed it was a binary classification task. It would be good to state that it is a 5-way classification task up front, rather than expecting the reading to dig it out of the methods.

****RESPONSE:** Thank you for this feedback. We have revised the manuscript to make it clear that our tasks involve a 5-way classification (lines 121 – 123)

- Were the training trials identical to the test trials (except for the provision of supervision?).

****RESPONSE:** The procedure for training and test trials was identical. We have clarified this in the Methods section as well as in the caption for the new Figure for Procedures (Fig 7). Based on feedback from Reviewer#2, we have also created a new Figure (Fig 2) illustrating the design of all the test trials.

- It would be good to know more details about the constraints on stimulus generation. The authors don't say how the different shapes were generated. How were they different from one another?

****RESPONSE:** All shapes consisted of six line-segments, one long and five short ones but the difference was in the relations between these line segments. There are, of course, many ways in which six line-segments can be organized, and we chose five shapes that, in our view, were clearly discernible from one another. We carried out a pilot study to monitor learning rate and observed that most participants could learn to distinguish these shapes within 300 trials. We have made this clear in the manuscript (lines 552 – 555).

- it would be good to know exactly what generalisation is over. Was it just position and rotation of the shape that varied?

****RESPONSE:** The generalization was over position, size and scale, as well as the exact shape. That is, we perturbed (within some bounds) the location and size of each patch within the overall shape, length of each segment and the location where segments intersect. During the test phase, new images differed in all these aspects and humans / CNNs had to generalise across these variations. We have clarified this in the text (lines 125 – 130).

- the authors say that the "location" of the colour patch varied. by location relative to what? presumably not in native space, because the shapes change position. relative to the segment? or the entire shape?

****RESPONSE:** We have clarified this in the revised text (line 125)

- more details are needed about the probabilistic model. How was its state space constructed? I couldn’t follow what exactly was done. In general, it might be useful to better motivate this model, which is not really discussed in detail.

****RESPONSE:** We have better motivated the statistical model in the Introduction and added a paragraph in the Methods section describing the intuition behind the computations performed by the model (lines 690 – 706). We have also added a paragraph clarifying the state-space over which the model operates for each Experiment (lines 738 – 753).

Reviewer #2:

Summary

This article compares human categorization performance to a convolution neural network (CNN) and a Bayesian statistical model (Ideal Classifier; IC). The stimuli for classification were novel, colored shapes.

In experiments 1-4, classification (into 1 of 5 categories) was possible through a conjunction of virtual object shape information and one of (a) patch location (a part of the virtual object appearing in a particular location and with a particular color), (b) segment color (part of the virtual object having a particular color), (c) average size of the virtual object, and (d) the color of the entire virtual object. Each experiment was paired with a second part, in which classification, during training,

by shape information was only possible on 80% of trials, while the other cue to classification remained 100% predictive.

Classification accuracy following training was measured in 4 conditions (a) both cues valid, (b) shape information valid, but the non-shape cue in conflict (indicating a different category), (c) the shape cue valid and the non-shape cue non-informative, and (d) the shape cue non-informative and the non-shape cue valid. The pattern of results for human participants suggested a strong bias to categorize based on shape, even when, during training, shape was less predictive of category membership than the non-shape cue. Use of the non-shape cue was negligible, except when the non-shape cue was the color of the entire virtual object. Differently, the CNN performed in a manner indicating a bias to classify based on the non-shape cue (but see below). The IC generally performs as expected - classifying at near ceiling in all cases except when the non-shape cue is in conflict.

A CNN trained to have a shape bias classified in a similar manner, but with more sensitivity to shape information. This result remained, even when pre-classification layers of the network were frozen, presumably maintaining much of the pre-trained shape bias.

In a fifth experiment, the same general procedure was used, but now with the absence of shape as a cue to category membership. Here as well, participants failed to learn to categorize based on patch location or segment color, but did learn to categorize based on size and color. The CNN, predictably, learned to categorize in all of these cases.

A sixth experiment showed that, even when participants were instructed to use the non-shape cue, they still struggled to do so, supporting the hypothesis that limited cognitive resources underlies the bias to rely on global shape information, even when this strategy is suboptimal.

Review

The impressive performance of CNNs, as compared to humans, have led some researchers to claim that the underlying representations, in network and in human, may be similar. However, the sometimes strange strategies employed by CNNs (e.g, using background information to classify an object) suggest fundamental differences from humans. This paper investigates if, how, and why CNNs might have different classification strategies. The results are, to me, interesting and convincing. CNNs are biased toward classifying virtual objects based on local features, whereas humans are biased to classify virtual objects based on global shape. The authors show that these biases are robust and fit with a theory they advance (and some modest evidence they present) that the human strategy results from cognitive limitations that are (relatively) absent for the CNN.

In general, I think the paper can be accepted with minor revisions mostly related to clarification:

****RESPONSE.** Thank you – we very much appreciate the positive review.

1. It is unclear how the Conflict test trials are scored (e.g., in Figure 2) when the concept is introduced on page 5-6.

****RESPONSE:** Yes, this is a good point. We have scored the Conflict trials based on the category mapping indicated by the shape cue. This means, if the participants / model is categorising based on shape, their performance should be high, but if they are categorising based on the non-shape cue, their performance should be low (below chance). We have clarified this in the text (lines 163 – 166).

2. Some more care could be taken to describe the four test trial types more clearly. Nothing in the paper is wrong but describing what each is (and is not), placed in list form outside the flow of the text would be helpful.

****RESPONSE:** Thank you for this feedback. We have created a new figure (Fig 2 in the new version of the manuscript) that illustrates the four types of test trials.

3. In Figure 1 caption, "movies S2 and S2" appears to be a typo.

****RESPONSE:** Fixed.

4. It is unclear to me why the performance of the IC is so low for conflict cases in Experiment 1. Wouldn't the statistical properties of the training and testing sets, wrt classification, be the same as in Exps. 2, 3, and 4?

****RESPONSE:** Yes, this was one of the unexpected results of the statistical inference model. The reason is that there is indeed a difference between the statistical properties of the training set in Experiment 1 compared to Experiments 2, 3 and 4. In this experiment, an image with a diagnostic patch at one of the diagnostic locations contains two types of predictive information: (i) a diagnostic colour at one of the diagnostic locations, and (ii) white (background) patches at all the other diagnostic locations. These two signals together dominate the shape signal in Conflict trials. We have added a footnote (Footnote 1, p. 7) to clarify this issue.

5. It seems a priori obvious that humans would not learn the patch location cue without enormous amounts of training, and maybe not even then. It's not terribly surprising that, even when told the diagnostic cue, performance in the patch condition was at chance. What is surprising, however, is that performance in the segment condition was also at chance, even when the relationship between the cue and the categories was made apparent to the participants. Is there a better explanation for why participants were at chance in this condition, other than that they didn't understand what they were supposed to do? Given the instructions, as described in the procedure section, it's hard to understand why participants were at chance for this condition in Experiment 6.

****RESPONSE:** Our intuition is that participants fail in this task because of the cognitive resources required to perform this task. For example, one strategy to perform the task would be to retain in memory the colour of the six segments on a trial and compare these colours to the colours of the six

segments on another trial which belongs to the same category. To follow this strategy, the participant needs to retain in memory the mapping between six colour and a category for each trial and find the intersection over trial that may or may not be consecutive. This obviously requires a lot of memory resources. Another strategy would be to randomly assign one of the (six) coloured segments to a category and check whether this mapping holds for another stimulus of the same category. This again requires a lot of trial and error over trials, again required memory and computational resources. We have added a paragraph to the Discussion (lines 503 – 517) to clarify these intuitions.

6. Swap is used instead of Conflict in some appendix figure captions

****RESPONSE:** Thank you for picking this up. We have corrected this.

7. A diagram of the experimental procedure (for humans) is always, IMO, helpful.

****RESPONSE:** Sure – we have added Fig 7 (p. 17) showing the experimental procedure to the Methods section.

Reviewer #3:

In this study, Malhotra et al., primarily probed the differences between CNNs and humans based on behavioral comparisons done with categorization tasks on images that were synthesized by varying specific image features. They conclude that these results highlight a fundamental difference between statistical inference models and humans.

Although the authors perform multiple experiments and several comparisons between CNN behavior and human behavior, it is very unclear to me what the central claim (or novel result) of the paper really is. Is it mainly that current CNNs are not fully predictive of human behavior (under the current specific training diet, architectural choices, learning objectives etc.)? If so, that point has already been made in multiple studies — and also not quantitatively assessed (with appropriate noise ceilings) in this study. Are the measurements done in this study better to falsify certain models or discriminate among similarly performing models than those done in earlier studies? It is not clear.

****RESPONSE:** We agree that there have been other studies that have made the point that current CNNs are not fully predictive of human behaviour. However, the question is how this behaviour is different. Past studies have focused on the training data, highlighting that CNNs trained on ImageNet are biased towards classifying objects based on textures (Geirhos et al) and that, when trained on the correct dataset, they can classify based on shape (Geirhos et al, 2018; Feinman & Lake, 2018; Hermann et al, 2020). In other words, a shape-bias in CNNs can be *acquired* from the training environment. While this may be true for human infants as well, we show here that CNNs and humans differ in how they select features in a new training set of unfamiliar objects. While human shape-bias "persists" (humans

continue to categorise object based on shape), CNNs are more flexible (they select whatever feature is most diagnostic). Thus, the dataset developed by us can indeed be used to falsify some models (e.g., a model that has been pre-trained on a Style-transfer ImageNet developed by Geirhos et al), which would not have been falsified by previous testing.

Our paper is not limited to these findings. We also show that when multiple global features are available, either of which can be used by participant for categorisation when presented on its own, participants usually select one of these features and ignore the other (Experiment 4 & Appendix B). This is again a feature of human behaviour that a model must replicate. We also show that people are unable to learn based on some cues (such as patch location or segment colour), even when these are pointed out (Experiments 5 & 6). This shows that human limitation in feature selection is not limited to feature discovery. We have revised our Introduction and Discussion to clarify these contributions.

The experiments (in this study), in my view were done (and presented) as to provide some insights into why such differences (between CNNs and humans) might exist. I have listed my major concerns with that approach below.

In line 68, the authors explicitly state that "Our objective was to see whether human inferences match the statistical inferences predicted by the ideal inference model and the CNN".

First, to make these comparisons, certain very basic premises need to be set. Other than differences in training of CNNs vs humans, it is critical in studies where CNNs and humans are compared to make specific (well grounded) commitments as to exactly how the CNNs are treated as primate vision models. What is the size of the input stimuli (that is field of view)? How long are the stimuli shown (i.e., is it rapid, "one-pass" for humans like in CNNs or long enough to engage recurrence)? Which layer is mapped onto to what brain area or what assumptions are made for the behavioral readout algorithms? All of these have been carefully explored in many different studies. Without establishing some very basic commitments, it is hard to evaluate the results of such studies with respect to the larger state of the field. Please see details below:

Field of view: What are the size of the presented images in visual degrees (for humans)? The authors state, "For the behavioural experiments, the stimuli size was scaled to 90% of the screen height (e.g. if the screen resolution was 1920x1080 the image size would have been 972x972). This ensured that participants could clearly discern the smallest feature in an image (a single patch) which we confirmed in a pilot study". That way of defining image size is not consistent with other studies and with usual regimes of images sizes (8 to 10 visual degrees) where the CNNs best predict human behavior.

****RESPONSE:** In an ideal world, we would have liked to carry out our study in the lab. But this has not been possible over the last two years. To conduct the study online, we had to work out what aspects of the stimuli are crucial for our investigation. To ensure that our results were not due to the limitations of visual acuity, we re-scaled stimuli to each participant's screen, ruled out participants under a certain

screen size and ran a pilot study to ensure that participants could discriminate colour and location of each patch.

However, we agree with the reviewer that ideally it would be good to test whether our results hold when the stimuli fall within a certain field of view. In order to check this, we have now found a way to rescale the stimuli online, based on the participant's screen size, calibrating the participant's screen and performing some checks. So, we have now carried out three new experiments (Experiments S1, S2, S3) where the stimuli were rescaled so that they always fell within 10 visual degrees for all participants. In these experiments we focused on the two types of non-shape cues, Patch location and Segment colour, where the participant behaviour differed most significantly from CNNs and the non-shape cue was either the stronger statistical signal (Experiments S1 and S2) or the only statistical signal (Experiment S3) that could be used for learning the task.

Results for these experiments are shown in Figure S7 (Appendix D). Participants showed the exact same pattern of results as our previous experiments. Their performance was statistically identical in the Same, Conflict and Shape conditions. When the shape cue was absent (Non-shape condition), their performance was at chance. Thus, these results replicate our previous findings and show that our results hold when stimuli fall within a certain field of view.

Presentation time: Most experiments in this study were run with 1000 ms to 3000 ms image presentations. These are timescales when various recurrent computations come into play (that are missing in current CNNs, shown across multiple research papers). The current CNNs are best approximations of early responses of the brain and the behavior that is also tested at those timescales.

****RESPONSE:** The reason to present stimuli for 1000ms – 3000ms was to give participants as much time as needed to learn the non-shape stimuli. Our hypothesis was that if participants do not pick a non-shape cue (such as patch location or segment colour) at this presentation time, they are unlikely to pick it at a faster presentation time.

However, the reviewer again makes a good point – most comparisons between humans and CNNs are made when the presentation rate is significantly faster, to avoid recurrent processes. Therefore, in the new set of experiments, we decreased the presentation time to 100ms. We also increased the number of trials to 450 (previous experiments used 300 trials) to give participants more opportunity to learn the cues. As mentioned above, we replicated all the patterns of results at this low presentation time. Participants continued to classify the stimuli based on shape even when shape was not the most predictive feature and when a coherent shape was not present, participants struggled to learn the task entirely.

Layer mapping: The fine-tuning of the model was done based on the penultimate layer of the model. It is not always the case that these are the best layers to predict human behavior (or inferior temporal cortex representations). In the Discussion, the authors mention: "These results pose a challenge for the hypothesis that humans and CNNs have similar internal representations of visual objects." This was a purely behavioral study and no concrete claims about any match between "internal

representations" of CNNs and humans should be made. There were no test/comparison of internal representations in this study. So such conclusions are not supported by data from this study. From this study, only a behavioral comparison can be made between these two systems (CNNs and humans).

****RESPONSE:** We think that it is unlikely that the CNN represents the global shape of a test stimulus, in addition to the non-shape diagnostic feature, given that when the CNN is tested only on global shape it's performance drops to chance (Experiments 1b, 3a, 3b, 4a & 4b) or near chance (all other experiments). However, we agree that it is possible that the global shape is represented in an internal layer, but simply never used for classification. What we want to emphasise is how this behaviour contrasts with human participants. We have therefore revised our Introduction and Discussion to reflect this and removed the sentence about internal representations that the reviewer mentioned.

Second, this specific question (do CNNs and humans make different inferences) has been asked multiple times before. It has been repeatedly tested before (in other studies) and most (if not all) models have been already falsified under various metrics (the authors cite some of those studies). Therefore an additional value that such a study can bring is if they provide a precise strategy (a training image-set, constraining data, or specific architectural insights) to develop better models of human vision or provide computationally explicit guidance. Unfortunately, in my opinion, the current study provides neither of these in a quantifiable or model-implementable way.

****RESPONSE:** The reviewer is correct – a number of studies have pointed out differences in inferences made by CNNs and humans and we have cited this work. However, we would also like to point out that there are a number of studies that have simultaneously made (and continue to make) the point that CNNs are good models of human vision. Here are some quotes from some recent studies published in leading journals:

"Deep neural networks provide the current best models of visual information processing in the primate brain" (Mehrer et al., 2021, PNAS)

"Primates show remarkable ability to recognize objects. This ability is achieved by their ventral visual stream, multiple hierarchically interconnected brain areas. The best quantitative models of these areas are deep neural networks..." (Zhuang et al., 2021, PNAS).

"...in recent years, a large body of work... has found that modern convolutional neural networks trained on image classification develop representations that match those found in the ventral stream. Early CNN layers match primary visual cortex (V1), while higher- level layers better match higher-level ventral stream areas, both in terms of qualitative preferred features [12] and quantitative predictions of responses to arbitrary stimuli [13, 14]." (Mineault et al., 2021, NeurIPS)

"...the representations of abstract shape similarity are highly comparable between macaque IT neurons and deep convolutional layers of CNNs that were trained to classify natural images, while human shape similarity judgments correlate better with the deepest layers" (Kalfas et al, 2018, PLOS Comp Bio)

“As a whole, [our] results indicate that convolutional neural networks not only learn physically correct representations of object categories but also develop perceptually accurate representational spaces of shapes.” (Kubilius, Bracci & Op de Beeck, 2016, PLOS Comp Bio)

There are many others. In light of these assertions, we believe it is important to also point out how the information processing in CNNs differs from human vision. While previous studies (such as Geirhos et al, 2018) have pointed out important differences, such as the lack of shape-bias, studies (including Geirhos et al themselves) have also suggested that one solution to this problem may be to train CNNs on a different dataset, rather than ImageNet. But this debate neglects how shape-bias affects learning of novel categories, which is what we focus on in our study. Our results show that the nature of shape-bias is qualitatively different in humans and CNNs. While the studies quoted above may be using a certain metric for asserting that CNNs are the “current best models” of human visual processing, our study provides an important behavioural phenomenon that must be captured by these models to be *good* models of human vision. We have edited our Introduction as well as Discussion to clarify these arguments.

The authors make the claim that differences between CNN and humans may be due differences in training environment. They claim that “To overcome these limitations, we conducted a series of experiments and simulations where humans and models had no prior experience with the stimuli.” --- There is no way to simulate a real "zero experience" for humans given that the human visual system is trained via evolution and development. Humans (as a species) likely have more than a lifetime of experience with objects that is reflected in their neural representations. They do not in all likelihood start with a blank slate. It is very difficult to make that assumption for humans, and the controls that the authors have done are certainly not complete. It is for the same reason that most studies only test the inference capability of trained CNNs (a “learned system”) when it comes to comparing CNNs with humans.

****RESPONSE:** Perhaps we did not motivate our choice of dataset clearly. Our intention was not to simulate a “zero experience” for humans. However, when comparing humans to CNNs, we could either compare their classification on known categories, such as dogs / cats / airplanes, etc. or novel categories that humans hadn’t experiences before. The reason not to choose the known categories was that humans have had a lifetime of experience dealing with these categories and different participants have had different experiences. Therefore, it made sense to compare humans and CNNs on a task where the categories are novel. The design of our stimuli (with patches of random sizes and colours ensures this). But this is not to say that humans have “zero experience” in dealing with these stimuli. Indeed, they bring their biases acquired over lifetimes (and, as the reviewer points out, over evolution) to the lab setting. The question we wanted to ask was whether training CNNs (on ImageNet or other datasets specifically designed to induce a shape-bias) can emulate the experience that humans bring to the lab. We have revised the Introduction and Discussion to clarify this issue.

The authors also claim that, “participant behaviour is better explained by a “satisficing” account [53] than an optimising account of object recognition”.

And, “Instead of an optimisation approach that underlies many machine learning models, we argue that human behaviour is much more in line with a satisficing account [53], where features are selected because they allow participants to perform reasonably on the task while taking into account their limited cognitive resources. While performing statistical inferences is certainly important, models of vision must also consider the cognitive costs and biases in order to be realistic theories of human object recognition.”

How do they measure the “limited cognitive resources”? Can that be tested in models or clearly defined and measured in humans for this study and the specific comparisons made? These words need to be operationalized as clear, falsifiable model components if they are used as speculations for mechanisms. Also, other words like “computational cost”, “cognitive capacities” etc. have been used for CNNs and humans without any concrete measurement or operationalization for most (if any) of them.

****RESPONSE:** At this stage we cannot offer specific operational definitions regarding the limited cognitive resources that may have played a role in our results. Our findings highlight an important disconnect between human and CNN vision, and at present, some sort of resource limitation is our best hypothesis. Clearly more research will be required before more detailed predictions can be made regarding whether indeed resource limitation is the explanation, and if so, what exactly the limitation is (are). We have clarified in the revised Discussion why we think human resource constraints may be preventing them from learning certain features, such as a single patch or a diagnostic segment (lines 503 – 525).

Some more specific comments:

51— Authors claim “while both system have the same learning objective” — that is a hypothesis/speculation and not a fact. So it should be written as such.

****RESPONSE:** We have deleted this line from the Introduction.

220- “If CNNs and humans are driven by performing statistical inferences, we expect both systems to start relying on the feature that is better at predicting the category label.” — This is a product of merging the learning and inference ideas. You can have two models where the internal representations can be the product of optimization on a very specific objective. But now when you take these representation to do another specific task — you can readout the behavior in two completely different ways (i.e., two different decoding models on same feature sets) to come up with massive mismatches in their output performance patterns. What the authors are trying to claim, cannot be tested unless they are really comparing the internal representations of CNNs with some neural data from humans.

**RESPONSE: We have revised this line to: “If the nature of shape-bias is similar in humans and CNNs, we expect both systems will adapt in a similar way to a new statistical environment, which favours a non-shape feature.” (line 253 – 255)

605 - “Since participants had a lifetime experience of classifying naturalistic objects prior to the experiment, we pre-trained our networks on a set of naturalistic images (ImageNet).” — This pre-training can also be assumed as the simulation of evolution instead of learning during the lifetime. The fine-tuning that follows after this — is a more likely substitute for learning during lifetime.

**RESPONSE: We have reframed this line as: “Since evolution and learning both play a role in how the human visual system classifies natural objects, we used a network that was pre-trained on naturalistic images (ImageNet) rather than trained from scratch. However, we observed the same pattern of results for a network that was trained from scratch.” (lines 669 – 673).

References:

Reuben Feinman and Brenden M Lake. Learning inductive biases with simple neural networks. arXiv preprint arXiv:1802.02745, 2018

Robert Geirhos, Patricia Rubisch, Claudio Michaelis, Matthias Bethge, Felix A Wichmann, and Wieland Brendel. Imagenet-trained cnns are biased towards texture; increasing shape bias improves accuracy and robustness. arXiv preprintarXiv:1811.12231, 2018.

Katherine Hermann, Ting Chen, and Simon Kornblith. The origins and prevalence of texture bias in convolutional neural networks. Advances in Neural Information Processing Systems, 33, 2020.

Harold Pashler and Michael C Mozer. When does fading enhance perceptual category learning? Journal of Experimental Psychology: Learning, Memory, and Cognition, 39(4):1162, 2013.

Larissa K Samuelson. Statistical regularities in vocabulary guide languageacquisition in connectionist models and 15-20-month-olds. Developmental psychology, 38(6):1016, 2002.

Nicolas W Schuck, Robert Gaschler, Dorit Wenke, Jakob Heinzle, Peter A Frensch, John-Dylan Haynes, and Carlo Reverberi. Medial prefrontal cortex predicts internally driven strategy shifts. Neuron, 86(1):331–340, 2015.

Linda B Smith, Susan S Jones, Barbara Landau, Lisa Gershkoff-Stowe, and Larissa Samuelson. Object name learning provides on-the-job training for attention. Psychological science, 13(1):13–19, 2002
